# Influencing factors of quality of life among front-line nurses who collected nucleic acid samples during COVID-19: a path analysis

**DOI:** 10.3389/fpubh.2023.1154725

**Published:** 2023-07-10

**Authors:** Jiaran Yan, Chao Wu, Yu Liu, Hongli Zhang, Chunyan He, Yawei Lin, Yang Li, Yuhai Zhang, Yinglan Li, Hongjuan Lang

**Affiliations:** ^1^School of Nursing, Air Force Medical University, Xi'an, Shaanxi, China; ^2^Xijing Hospital, Air Force Medical University, Xi'an, Shaanxi, China; ^3^School of Nursing, Shaanxi University of Chinese Medicine, Xianyang, Shaanxi, China; ^4^Department of Health Statistics, Air Force Medical University, Xi'an, Shaanxi, China; ^5^Xiangya Nursing School, Central South University, Changsha, Hunan, China

**Keywords:** quality of life, influencing factors, COVID-19, nurses, public health

## Abstract

**Aim:**

The aim of this study is to investigate the quality of life of nurses who collected nucleic acid samples throughout the COVID-19 epidemic's routine management, as well as the factors that may have influenced it.

**Background:**

After the outbreak of COVID-19, normalized epidemic prevention and control throughout China were implemented. Nucleic acid testing has become an effective measure for the early detection of virus-infected individuals. Nurses collecting nucleic acid samples undertake important tasks. Their quality of life is significant to maintaining team stability and containing the epidemic. However, research on their quality of life is still limited.

**Methods:**

A cross-sectional survey was conducted on 1,292 nurses who collected nucleic acid samples from five tertiary general hospitals in Xi'an through self-reported electronic questionnaires (including general demographic information, the Connor–Davidson Resilience Scale, the Social Support Rating Scale, and the World Health Organization Quality of Life Brief Scale). Descriptive, one-way ANOVA and multiple linear regression were performed using SPSS 26.0. Structural equation modeling was used to analyze the influencing factors.

**Results:**

The nurses collecting nucleic acid samples had a modest level of quality of life. Age, marital status, average daily sleep duration, frequency of exercise, psychological resilience, and social support were all influencing factors of quality of life, according to multiple linear regression analysis. Quality of life was found to be significantly related to psychological resilience and social support.

**Conclusion:**

Demographic characteristics, psychological resilience, and social support are the factors affecting the quality of life of nurses who collect nucleic acid samples. Nursing managers should focus more on these factors to improve the quality of life for nurses.

**Relevance to clinical practice:**

Nursing managers should realize the importance of the quality of life of nurses who collect nucleic acid samples in maintaining a vigorous nursing team and ensuring optimized epidemic control. Social support should also be provided to nurses to improve their psychological resilience, thereby improving their quality of life.

## 1. Introduction

The medical personnel working on the front lines of the pandemic have faced challenging testing since the COVID-19 outbreak (1), which has increased the incidence of psychological crises and affected people's physical health (2–4). Ensuring the physical and mental health of medical staff is an important issue in the battle against this epidemic. At present, the spread of COVID-19 in China is well under control. However, sporadic cases, as a result of the strong virus mutation, vigorous infectivity, and increased incubation time, as well as the presence of asymptomatic infections, have prolonged the epidemic (5, 6). This causes significant disruptions in our everyday lives and presents difficulties for the clinical medical staff. In the case of the COVID-19 outbreak in Xi'an, China, at the end of 2021, the city was under routine control and monitored for epidemics during the outbreak. During these days, regular national nucleic acid testing has become a key strategy for preventing and controlling epidemics (5).

The front-line nurses who collected nucleic acid samples are the main contributors to this process; they are under a lot of workload and pressure at work. Compared with doctors, nurses report lower levels of job satisfaction and higher levels of stress and despair (7). Their quality of life affects patient safety and quality of care (8). Poor quality of life will make nurses less motivated to work and more burned out on the job. It can easily result in brain drain and is detrimental to the stability and growth of the nursing team. Therefore, it is crucial to consider the nurses' quality of life.

Numerous studies on the quality of life of nurses have been conducted during the COVID-19 outbreak (9, 10). For example, a study by Nishi Suryavanshi et al. (11) found that during the COVID-19 pandemic, the prevalence of depressive and anxiety symptoms among Indian healthcare workers was high, with 45% of healthcare workers reporting below-average quality of life. According to the research by Ying An et al., depression among emergency department nurses was widespread during the COVID-19 pandemic, with a prevalence rate of 43.61% overall. Depressed nurses also reported lower quality of life than non-depressed nurses (12). A study by Yadollahpour et al. (13) found that 69% of nurses at a university hospital in Iran experienced moderate occupational stress and moderate quality of life during the COVID-19 pandemic. In addition, nurses perceived adequate income as a positive predictor of quality of life. According to the research by Liang et al. medical staff experienced negative emotions such as anxiety and depression, during the COVID-19 epidemic (14, 15), which had a negative impact on nurses' quality of life. However, according to research by Nashwan et al. (16), the quality of life of nurses in Qatar is on a positive level, irrespective of whether they were assigned to a COVID-19 institution.

Nurses who collect nucleic acid samples are recruited from different clinical departments. Their workload increased, as they not only had to complete clinical work but also undertake nucleic acid collection work for a large number of people. A study by Zhu et al. found that long-term use of protective equipment significantly increased the psychological and physical discomfort of the nurses taking nucleic acid samples (17). However, few research studies have been conducted on the quality of life of front-line nurses who collect nucleic acid samples, especially during the period of routine epidemic control. Therefore, this study aims to investigate the quality of life of front-line nurses participating in nucleic acid sample collection and its influencing factors under the normalized management of the epidemic, to provide a theoretical basis for improving their quality of life. It will be significant in stabilizing the nurse talent team and epidemic prevention and control.

### 1.1. Background

The World Health Organization's (WHO) new concept of health is used to conceptualize quality of life. WHO defines the quality of life (QOL) as follows: the experience of individuals in different cultural systems and value systems about their life goals, expectations, standards, and concerns about their living conditions (18). Quality of life is a key indicator of individual wellbeing and perception of life experiences (19). The quality of life of nurses has a direct impact on the efficiency and standard of their work (8). Nurses' enthusiasm and job satisfaction can both increase with a high quality of life.

Psychological resilience is the capacity to recover from negative experiences. It is described as a person's capacity to deal with adversity, threat, or other challenging circumstances (20). It can shield people from the negative impacts of adversity. It is critical in enhancing mental health and can help people deal with challenging circumstances (21, 22). Higher psychological resilience is beneficial for individuals to find a positive way to deal with stressful situations (23). It is conducive to increasing work enthusiasm and satisfaction and also can alleviate the negative impact of job burnout (24). It can also mitigate the negative effects of perceived stress on insomnia (25) and help to improve sleep quality, which is essential to the overall quality of life.

Social support is defined as the material or spiritual aids that people receive from friends, family, and other people in stressful situations, and it helps improve people's mental health (26). Social support represents an external protective force. According to Nie et al. (27), feeling more socially supported can lessen psychological suffering and change people's perception of stress (28), thereby assisting people in keeping their emotions in check when under pressure (29). According to Xiao et al., extensive social support improves self-efficacy (30). Social support is considered the primary factor that maintains one's physical and mental health, enhancing their quality of life (31). Adequate social support is helpful for nurses to relieve anxiety and tension, increase passion for their work and lives, and therefore reduce the turnover rate of nurses (32). Lee et al.'s research showed that social support is one of the important factors affecting nurses' health promotion behavior (33), and adequate social support can improve the wellbeing of life.

### 1.2. Aim

This study is aimed at investigating (1) the quality of life, psychological resilience, and social support of front-line nurses collecting nucleic acid samples; (2) the relationship between quality of life, psychological resilience, and social support; and (3) the factors that influence nurses' quality of life and the structural model.

## 2. Methods

### 2.1. Study design

A cross-sectional survey was conducted from May to June 2022. This survey adopted the convenient sampling method to select front-line nurses who collected nucleic acid samples from five tertiary general hospitals in Xi'an, Shaanxi, China, as the survey objects. Nurses completed the survey through a self-reported online questionnaire.

### 2.2. Sample and setting

The inclusion criteria for nurses were as follows: (1) possession of a People's Republic of China nurse qualification certificate; (2) participation in at least one nucleic acid collection during the COVID-19 epidemic; and (3) informed consent and voluntary participation in the study. We excluded nurses who were on leave or studying. The sample size was calculated based on 10 times the number of scale entries. This questionnaire contains 71 items, and the formula for calculating the sample size is *N* = (10 + 25 + 10 + 26)^*^ 10 = 710, which means that this study needed at least 710 participants (34). A total of 1,292 nurses who collected nucleic acid samples were enrolled in Xi'an. Ultimately, 1,224 valid questionnaires were collected with an effective response rate of 94.74% (68 questionnaires with incomplete responses).

### 2.3. Measures

#### 2.3.1. Demographic information questionnaire

The questionnaire was developed by the researchers based on a literature review and expert consultation. It contained a total of 10 items, including gender, age, education level, professional title, marital status, years of work, monthly income, average daily sleep duration, frequency of exercise per week, and the number of night shifts per month.

#### 2.3.2. Psychological resilience

Psychological resilience was measured using the Chinese version of the Connor–Davidson Resilience Scale (CD-RISC) (35), which was translated and revised into Chinese by Yu Xiao-Nan and Zhang Jian-Xin (36). The CD-RISC comprises 25 items across three subdomains: strength (eight items), tenacity (thirteen items), and optimism (four items). The participating nurses responded to the items on a five-point Likert scale ranging from 0 (“never”) to 4 (“almost always”). The total psychological resilience score ranged from 0 to 100. The higher the score, the more psychological resilience there is. Cronbach's alpha for this scale in this study was 0.943.

#### 2.3.3. Social support rating scale

Social support was measured using the Chinese version of the Social Support Rating Scale (SSRS), which was translated and developed by Xiao Shui-Yuan (37). The SSRS consists of 10 items across three subdomains: objective support (three items), subjective support (four items), and support utilization (three items). The options for items 1–4 and 8–10 are graded from “1” to “4.” Item 5 is scored as A, B, C, and D, with each item ranging from “none” to “full support” from “1” to “4,” respectively. The score for questions 6 and 7 depends on the number of support sources selected. A higher score indicates greater social support. Cronbach's alpha for this scale in this study was 0.705.

#### 2.3.4. Quality of life

Quality of life was measured using the Chinese version of the World Health Organization Quality of Life Brief Scale (WHOQOL-BREF) (38), which was translated and revised by Fang et al. (39). The WHOQOL-BREF comprises 26 items. Questions 1 and 2 are independent items used to measure an individual's general subjective quality of life and physical wellbeing. The remaining 24 items are divided into four dimensions: physical (seven items), psychological (six items), social relationship (three items), and environment (eight items). The nurses responded to the items on a five-point Likert scale ranging from 1 (“strongly agree”) to 5 (“strongly disagree”). The average score of the listed items is multiplied by 4 to determine the score for each dimension. The score of quality of life is calculated by totaling the four dimensions, with higher scores suggesting a better quality of life (40, 41). Cronbach's alpha for this scale in this study was 0.936.

### 2.4. Ethical consideration

The study is guided by the Declaration of Helsinki's code of ethics and the institution's ethical standards. The study was presented to the Ethics Committee of the Xijing Hospital of Air Force Medical University and did not involve immoral behavior (No. KY20224143-1). The first section of the questionnaire is the informed consent, which participants can accept or reject before moving on. The survey is anonymous and maintains the privacy of personal information. The decision of whether or not to answer the questionnaire is up to the participant. In our study, informed consent was obtained from all the participants.

### 2.5. Data collection

An electronic questionnaire was designed to collect the data from the online survey through WeChat. Hospital administrators and department heads were briefed on the goals and significance of the study, and their support was gained. The electronic questionnaire was filled out by the nurses who collected the nucleic acid samples with assistance from the head nurse. In the introduction, a promise that the data will be kept private and used only for that research is made, along with an explanation of the research's purpose and detailed methods of implementation. Second, the follow-up questionnaire could only be completed if the nurses confirmed that they were voluntarily participating on an informed consent form.

### 2.6. Data analysis and availability

The data were processed with IBM SPSS version 26.0 after being entered into an excel sheet. Frequency and composition ratio were used to describe the general state of the participants. The psychological resilience, social support, and quality of life scores of participants were denoted in terms of their mean, standard deviation (*SD*), and minimum and maximum values, as these data were normally distributed by the Shapiro–Wilk test. The influencing factors of nurses' quality of life were investigated using single-factor analysis (containing a *t*-test or analysis of variance [ANOVA]) and multivariate analysis (stepwise multiple regression analysis). The structural equation model (*SEM*) was utilized to investigate the influencing factors of quality of life, and MPLUS (version 8.3) was used to measure and establish the structural model. The maximum likelihood was used to estimate the parameters of the following model. The indexes of fitness of the *SEM* are as follows: The statistical measures used were the chi-square test (χ2; χ2/df <3.0), the comparative fit index (CFI ≥0.90), the Tucker–Lewis index (TLI ≥0.90), the root mean square error of approximation (RMSER ≤ 0.06), and the standardized root mean square residual (SRMR ≤ 0.05) (42). To determine the 95% confidence interval (CI) and corresponding significance of the effects, a bootstrapping sample of 1,000 was employed. Statistical significance was defined as a *P*-value of <0.05.

## 3. Results

### 3.1. Characteristics and distribution of nurses' quality of life

All of the respondents were women, with an average age of 31.41 years (*SD* = 5.69; 21–49 years) and an average of 8.94 working years (*SD* = 6.27; 6 months−30 years). They took part in the nucleic acid collection task for ~4 h each time and participated 2–3 times a week. A total of 620 respondents (50.7%) have middle titles, followed by 264 (21.6%) junior titles and 340 (27.8%) senior titles. In total, 863 responders (70.5%) were married, 345 (28.1%) were single, and 16 (1.3%) respondents were either divorced or widowed. The findings of a one-way ANOVA showed that the nurses' quality of life scores varied considerably depending on their age, professional title, marital status, working years, monthly income, average daily sleep duration, frequency of exercise, and the number of night shifts (*P* < 0.05). Table 1 shows the differences in demographic characteristics and quality of life scores of nurses who collected nucleic acid samples.

**Table 1:** The univariate analysis of general information and quality of life (*n* = 1, 224).

**Variable**	***n* (%)**	**Mean (*SD*)**	***F***	***P***
**Age (years)**			12.594	<0.001
<30	504 (41.2)	52.09 (8.57)		
30–39	601 (49.1)	51.48 (7.94)^d^		
≥40	119 (9.7)	55.58 (7.80)^b^		
**Educational level**			0.068	0.934
Junior college or below	131 (10.7)	51.96 (8.61)		
Undergraduate	1079 (88.2)	52.14 (8.28)		
Master degree or above	14 (1.1)	52.76 (4.12)		
**Professional title**			13.353	<0.001
Junior	264 (21.6)	52.75 (8.15)		
Middle	620 (50.7)	50.98 (8.27)^a^		
Senior	340 (27.8)	53.74 (8.07)^d^		
**Marital status**			4.629	0.010
Unmarried	345 (28.2)	50.99 (8.55)		
Married	863 (70.5)	52.57 (8.13)^a^		
Divorced or widowed	16 (1.3)	52.86 (8.29)		
**Years of working**			10.966	<0.001
<5	262 (21.4)	52.26 (8.02)		
5–10	622 (50.7)	51.20 (8.46)		
11–20	255 (20.8)	52.84 (7.74)^d^		
>20	85 (7.0)	56.36 (7.80)^c^^f^		
**Monthly income (RMB)**			7.700	<0.001
≤ 3,000	80 (6.5)	52.56 (7.75)		
3,001–5,000	181 (14.8)	49.94 (8.74)^a^		
5,001–8,000	654 (53.4)	51.99 (8.35)^d^		
≥8,001	309 (25.2)	53.59 (7.67)^e^^f^		
**Average daily sleep duration (hours)**			21.746	<0.001
<6	411 (33.6)	50.04 (8.44)		
6–8	778 (63.6)	53.08 (7.82)^a^		
>8	35 (2.9)	55.46 (11.06)^b^		
**Frequency of exercise per week**			28.192	<0.001
0	768 (62.7)	50.85 (8.07)		
1–2	388 (31.7)	53.94 (7.96)^a^		
≥3	68 (5.6)	56.26 (9.05)^b^		
**Number of night shifts per month**			8.690	<0.001
0	305 (24.9)	54.14 (7.30)		
1–3	157 (12.8)	51.83 (7.86)^a^		
4–6	594 (48.5)	51.54 (8.79)^b^		
≥7	168 (13.7)	50.81 (7.86)^c^		

a Comparison of the first and second items.

b Comparison of the first and third items.

c Comparison of the first and fourth items.

d Comparison of the second and third items.

e Comparison of the second and fourth items.

f Comparison of the third and fourth items (*P* < 0.05).

SD, standard deviation.

### 3.2. Psychological resilience, social support, and quality of life among nurses

The nurses for this study had a quality of life score of 52.13 (*SD* = 8.27), a psychological resilience score of 55.75 (*SD* = 14.96), and a social support score of 36.11 (*SD* = 7.86). Table 2 displays the precise scores for each dimension. The findings of the correlation study revealed a strong positive link between nurses' quality of life and social support (*r* = 0.522, *P* < 0.01) and psychological resilience (*r* = 0.590, *P* < 0.01). Each dimension of quality of life was significantly positively correlated with each dimension of psychological resilience and social support (*P* < 0.01). Table 3 shows the specific correlation coefficients.

**Table 2:** The scores of psychological resilience, social support, and quality of life.

**Scales**	**Minimum**	**Maximum**	**Score**	**Average score**
**Psychological resilience**	11.00	100.00	55.75 (14.96)	2.23 (0.06)
Strength	3.00	32.00	19.46 (5.00)	2.43 (0.63)
Tenacity	3.00	52.00	28.03 (8.49)	2.16 (0.65)
Optimism	1.00	16.00	8.27 (2.69)	2.07 (0.67)
**Social support**	16.00	57.00	36.11 (7.86)	3.61 (0.79)
Objective support	3.00	21.00	9.18 (3.31)	3.06 (1.10)
Subjective support	8.00	28.00	19.48 (4.47)	4.87 (1.12)
Support utilization	3.00	12.00	7.44 (1.87)	2.48 (0.62)
**Quality of life**	19.24	75.71	52.13 (8.27)	3.18 (0.99)
Physical	4.57	18.86	13.24 (1.96)	3.31 (0.49)
Psychological	5.33	19.33	13.16 (2.09)	3.29 (0.52)
Social relationship	4.00	20.00	13.49 (2.96)	3.37 (0.74)
Environment	4.00	20.00	12.46 (2.64)	3.12 (0.66)

**Table 3:** The correlation between quality of life, psychological resilience, and social support.

**Item**	**Quality of life**	**Physical**	**Psychological**	**Social relationship**	**Environment**
**Psychological resilience**	0.590^**^	0.504^**^	0.528^**^	0.488^**^	0.520^**^
Strength	0.571^**^	0.498^**^	0.522^**^	0.463^**^	0.497^**^
Tenacity	0.559^**^	0.479^**^	0.494^**^	0.469^**^	0.489^**^
Optimism	0.456^**^	0.364^**^	0.409^**^	0.374^**^	0.424^**^
**Social support**	0.522^**^	0.413^**^	0.429^**^	0.489^**^	0.450^**^
Objective support	0.369^**^	0.309^**^	0.303^**^	0.331^**^	0.321^**^
Subjective support	0.475^**^	0.377^**^	0.392^**^	0.458^**^	0.394^**^
Support utilization	0.406^**^	0.291^**^	0.329^**^	0.376^**^	0.382^**^

** *P* < 0.01.

### 3.3. Regression analysis of factors influencing quality of life

The dependent variable was the quality of life score, while the independent factors were the dimensions of resilience and social support, as well as demographic variables with statistically significant differences in a one-way ANOVA. The dimensions of resilience and social support are independent variables.

Table 4 shows the results of the multiple linear regression analysis. The Durbin–Watson statistical value is close to 2 at 2.095, indicating that there is no sequence correlation. The results of the collinearity diagnosis show that the variance inflation factor (VIF) is <10, indicating that there is no multicollinearity. The results of multiple regression analysis showed that age (β = −1.003, *P* < 0.05), marital status (β_1_ = −2.025, *P* < 0.01; β_2_ = −3.335, *P* < 0.05), sleep duration (β_1_ = 1.331, *P* < 0.01; β_2_ = 3.616, *P* < 0.01), frequency of exercise (β_1_ = 2.130, *P* < 0.01; β_2_ = 0.994, *P* < 0.05), psychological strength (β = 0.452, *P* < 0.01), tenacity (β = 0.149, *P* < 0.01), objective support (β = 0.317, *P* < 0.01), subjective support (β = 0.458, *P* < 0.01), and support utilization (β = 0.406, *P* < 0.01) entered the regression equation of the total score of quality of life. The fitting equation was statistically significant according to the *F*-test results, which also showed that *F* = 76.840, *P* < 0.001; *R*^2^ = 0.471; and adjusted *R*^2^ = 0.465, which suggested that the independent variables explained 47.1% of the variance variability.

**Table 4:** Multiple linear regression analysis of quality of life (*n* = 1, 224).

**Independent variable**	**β**	**95%*CI***	***P***
Constant	24.826	22.942 to 26.711	**<0.001**
**Age (years)**			
<30 (ref)			
30–39	−1.003	−1.859 to−0.148	**0.022**
≥40	−0.088	−1.436 to 1.261	0.898
**Marry status**			
Unmarried (ref)			
Married	−2.025	−3.043 to−1.007	**<** **0.001**
Divorced or widowed	−3.335	−6.489 to−0.181	**0.038**
**Average daily sleep duration (hours)**			
<6 (ref)			
6-8	1.331	0.590 to 2.072	**<** **0.001**
>8	3.616	1.506 to 5.726	**0.001**
**Frequency of exercise per week**			
0 (ref)			
1–2	2.130	0.579 to 3.681	**0.007**
≥3	0.994	0.236 to 1.752	**0.010**
**Psychological resilience**			
Strength	0.452	0.316 to 0.588	**<0.001**
Tenacity	0.149	0.074 to 0.224	**<0.001**
Optimism	0.010	−0.172 to 0.191	0.917
**Social support**			
Objective support	0.317	0.187 to 0.447	**<0.001**
Subjective support	0.458	0.354 to 0.562	**<0.001**
Support utilization	0.406	0.194 to 0.617	**<0.001**

Determination coefficient *R*^2^ = 0.471; adjusted determination coefficient *R*^2^ = 0.465.

Durbin-Watson = 2.095; *F* = 76.840 (*P* < 0.001).

The bold values indicate the value of *P* < 0.05, the difference is statistically significant.

### 3.4. Structural equation modeling for quality of life

**Figure 1:**
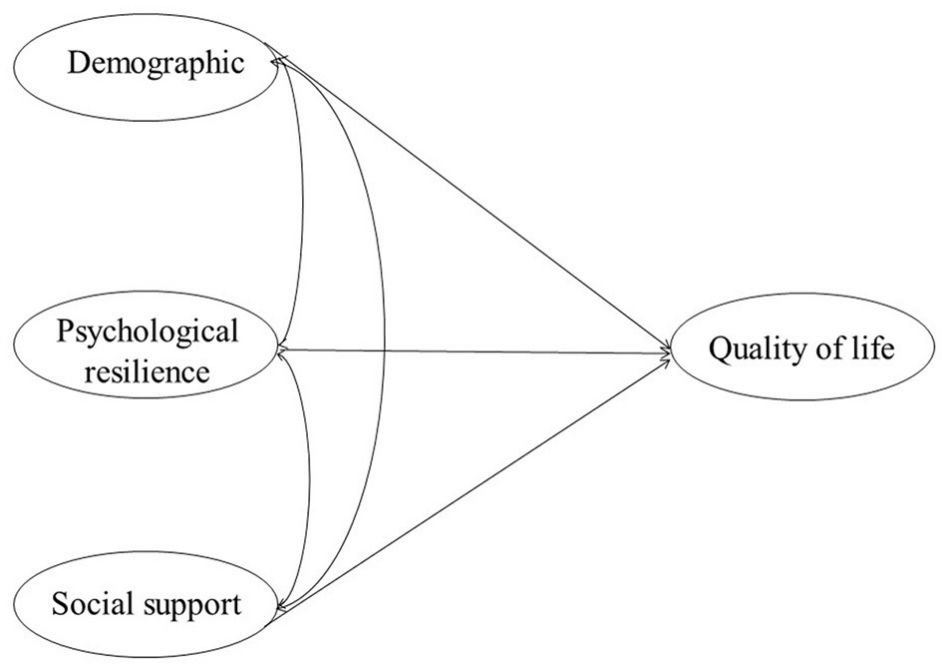
Hypothetical model of factors influencing nurses' quality of life.

**Figure 2:**
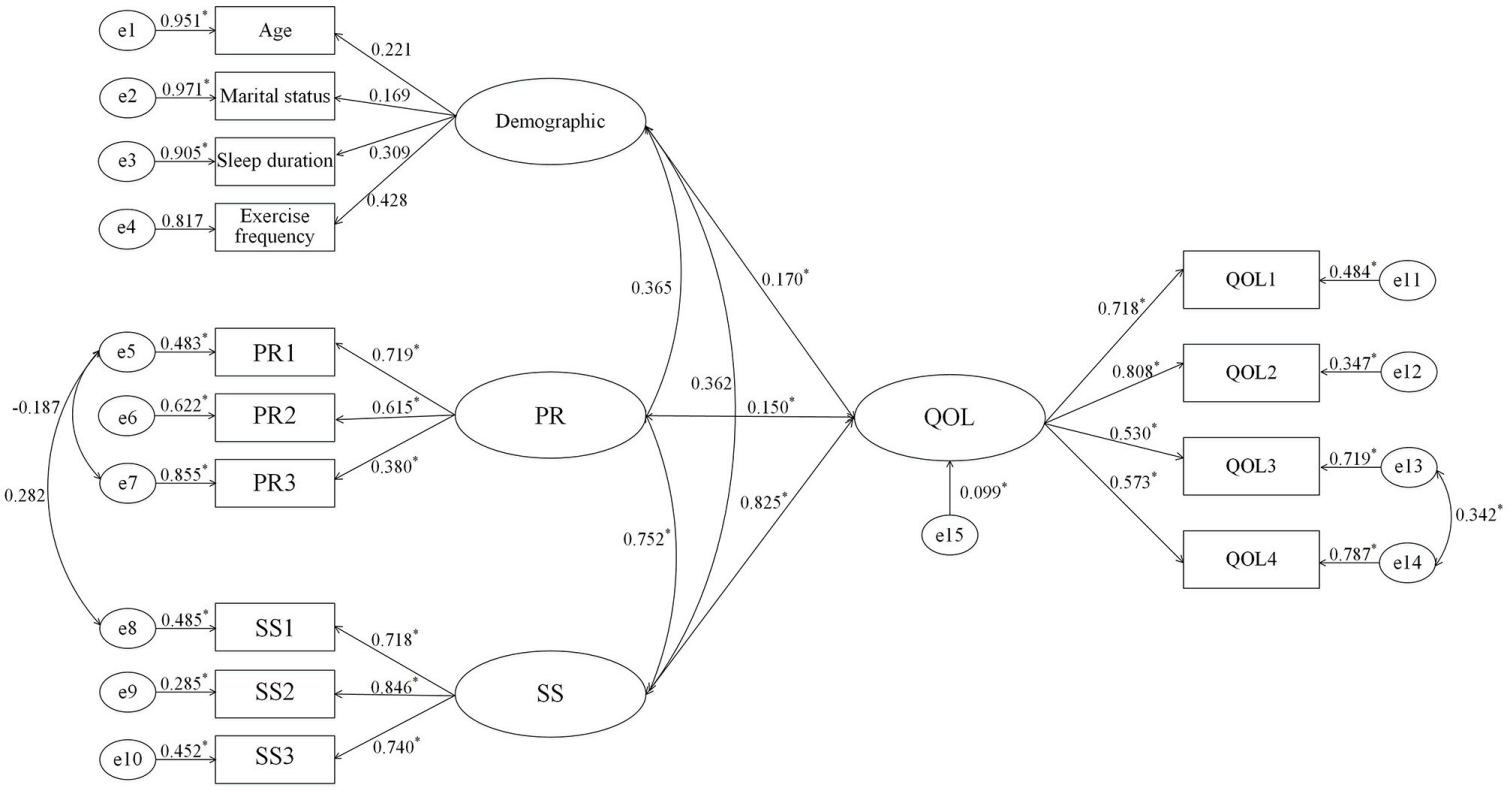
Path parameters of model. QOL, Quality of life; PR, Psychological resilience; SS, Social support; QOL1–QOL4, manifest variables of quality of life; PR1–PR3, manifest variables of psychological resilience; SS1–SS3, manifest variables of social support. ^*^P < 0.05.

We created a structural equation model of quality of life using the outcomes of the multiple linear regression analysis (Figure 1). To determine whether the measurement model's metrics fit the requirements, we performed a confirmatory factor analysis (Figure 2). The results were as follows: χ^2^/*df* = 2.92 (<3), confirmatory fit index (CFI) = 0.978 (≥0.90), Tucker–Lewis index (TLI) = 0.967 (≥0.90), standardized root mean square residual (SRMR) = 0.030 (≤ 0.05), root mean square error of approximation (RMSEA) = 0.040 (≤ 0.06). The model fits well, as shown by the fact that all fitting measures are within an acceptable range (42, 43). Demographics, psychological resilience, and social support significantly impacted the quality of life in structural equation modeling.

## 4. Discussion

### 4.1. The state of nurses' quality of life

The quality of life score for the nurses was 52.13 (*SD* = 8.27), which was not different from our previous score on the quality of life of nurses in the infectious disease departments (*t* = 1.417; *P* > 0.05), both of which were at the lower middle level (44). The following factors may contribute to these distinctions: in addition to performing the department's duties as usual, these nurses must also collect thousands of nucleic acid samples. The stress on nurses is increased by their heavy workload. Most nucleic acid collection sites are outdoors, with poor infrastructure, confined protective equipment, and high temperatures, which may make it uncomfortable for nurses to collect nucleic acid samples. In addition, since the COVID-19 epidemic began, there have been some reports of medical personnel infections (45). COVID-19 has a high level of variability, which increases infectivity while allowing for immunological escape. Nurses who collect nucleic acid samples have to deal with complex personnel every day, and there is a risk of infection. Some nurses have certain fears about this. These will have some effect on the quality of life of nurses.

### 4.2. How generic demographic features affect the overall quality of life

In general, demographic information, factors, including age, marital status, sleep duration, and frequency of exercise per week all have an effect on nurses' quality of life. The study indicated that nurses in the 30–39 age group had a slightly lower quality of life than those in the younger age group. The following are fundamental to the analysis: this age cohort of nurses increasingly becomes the department's backbone, with job promotion being a major stressor. The level of job burnout among nurses rises with age (46). In addition, as compared to younger nurses, they are less physically fit and are less able to tolerate wearing protective gear and clothing. Nursing management should develop incentive programs and provide acceptable opportunities for advancement in order to motivate nurses. In addition, the proportion of young nurses in the nucleic acid sample collection team can be appropriately increased. To fully understand their needs, a high level of attention should be paid to the physical and mental health of nurses in this age group.

Married nurses live less happily than their single counterparts. According to a study of Turkish healthcare professionals, married healthcare employees demonstrated significantly higher levels of stress, anxiety, and occupational burnout than unmarried employees (47). Most of the women in our survey are currently juggling parenthood and supporting their parents. Nurses feel stress, which reduces their quality of life. The unexpected onset of the epidemic, the unpredictability of working hours, the fear of contracting the disease and transmitting it to family members, and other factors all contribute to this stress (27, 48). The risk factors that affect the quality also include concurrent divorce and widowhood. Women who have experienced these things rarely receive help or support from their families, and they are unable to talk to them about their stress or troubles at work. Since there is only one person responsible for the family and kids, they feel stressed both financially and socially, which decreases their quality of life. Nursing management needs to be more conscious of the emotional and psychological changes they are experiencing. It is feasible to offer support and reassurance to nurses who are having family burdens and to alleviate the burden on their families, which may improve the nurses' quality of life and job satisfaction.

According to the study, increased daily average sleep time improves the quality of life. A good night's sleep allows people to recover from a day's work and has more energy to maintain a work–life balance. According to research, nurses who provide front-line care for COVID-19 patients frequently experience varying degrees of sleep disturbance (49). Many healthcare professionals struggle with insomnia (9). A study of Egyptian medical staff by Mohamed et al. (50) found that longer work hours and less sleep per week were associated with higher levels of health anxiety about COVID-19 infection among healthcare workers.

The task of collecting national nucleic acid samples adds to nurses' burden and working hours. Anxiety and despair can also result in sleep issues in nurses (51). According to Xiao et al. (32), getting enough quality sleep might have a positive impact on one's physical and mental health by alleviating their anxiety and stress levels (52). Nursing management should pay attention to the nurses' sleep habits and design reasonable and flexible shift schedules in order to guarantee that the nurses receive as much sleep as possible. Regular physical activity has a positive effect on the quality of life. According to research by Wang et al. (53) higher sleep quality and regular exercise have a beneficial impact on nurses' compassion satisfaction, which can make nurses feel good and devote more energy to work. Appropriate exercise can help people cope with stress and anxiety in addition to maintaining good physical health (54, 55). Nurses frequently skip workouts due to their hectic schedules, weariness, and lack of time. Hospital administrators could encourage nurses to exercise by building gyms or organizing group activities.

### 4.3. The effect of psychological resilience on quality of life

Psychological resilience is an influencing factor of quality of life and has a significant positive correlation with it, which is consistent with the findings of Atay et al. (56). The higher the psychological resilience, the better the quality of life. Nurses with strong psychological resilience can actively mobilize psychological resources and be more optimistic and courageous to face stressful situations and challenges in life. The psychological resilience score of nurses who collected nucleic acid samples in this study was 55.75 (*SD* = 14.96), which was significantly lower than that of Pan et al. (*t* = −19.902; *P* < 0.001) for the personnel of the central sterile supply department (57). In addition, it is lower than the findings of Zhang et al. (58) on clinical nurses in tertiary hospitals. Not all nurses who collect nucleic acid samples work in the infectious disease department. Most of the knowledge about the prevention and control of infectious diseases is acquired after short-term training. The higher the psychological resilience, the better the quality of life. Nurses with strong psychological resilience can actively mobilize psychological resources and be more optimistic and courageous to face stressful situations and challenges in life. Studies have shown that psychological resilience is closely related to work stress (59–61). Nurses will experience anxiety if their psychological resilience is insufficient to effectively relieve stress. Anxiety, depression, and stress are the determinants of life satisfaction (62), which are not conducive to the quality of life. Nursing managers should be cautious of nurses' levels of psychological resilience and regularly assess their mental health. Lectures on mental health knowledge and proper stress management can help nurses improve their psychological resilience, thereby enhancing their quality of life.

### 4.4. The impact of social support on quality of life

Social support is an important factor affecting the quality of life. It is positively correlated with quality of life and can positively predict nurses' quality of life, consistent with Jubin et al.'s findings (19). In addition, in line with the previous researchers' findings, social support is significantly positively correlated with psychological resilience, which is consistent with the study of Wang and Warshawski (23, 63). Good social support helps nurses to satisfy their physical, psychological, and professional needs. They can rely on their friends, family, and the community for assistance when facing problems or in need of support. Having respect and support from others can help people feel less stressed and anxious (32). Good social support can help people increase their sense of self-worth and self-confidence, psychological resilience, and wellbeing, all of which contribute to a nurse's quality of life. According to the study's results, nurses had a moderate social support score of 36.11 (*SD* = 7.86), which was substantially higher than that of Xiao et al. (32) (*t* = 8.615; *P* < 0.001). This may be due to factors such as the government's policy of giving front-line nurses preferential treatment and positive media publicity. The motivation and job satisfaction of nurses can be increased with enough organizational support. To encourage nurses to work, nursing management might create supporting incentive systems. The management can also foster positive interpersonal relationships by creating a positive work atmosphere in their department, actively planning team-building activities, and encouraging the nurses to support one another. The head of the department and seniors can have exchanges with nurses' families to help gain the family respect and support for the nurses' work. All of the foregoing should, in theory, improve nurses' quality of life.

## 5. Limitations and future research

There are some limitations to our research. To begin with, only the Xi'an nurses who collect nucleic acid samples are surveyed. The quality of life of the medical staff needs to be given more thought. Second, the convenience sampling approach was used for this study as it is chosen at the onset. Stratified random sampling will be used in future research, as it may generate more valid results, particularly in large-scale surveys. In the upcoming study, we will give interventions to nurses to see if their quality of life improves.

## 6. Relevance to clinical practice

Hospitals and nursing managers should be concerned with the life quality of nurses who collect nucleic acid samples. Efforts should be made to improve their life quality and work efficiency so that this nursing team can remain stable. Managers should emphasize the importance of psychological resilience and social support for nurses' quality of life. Lectures on positive psychology, meditation training (64), and stress management (65) should be provided to help nurses better cope with challenges and stress, effectively coordinate stressful life events, and improve psychological resilience (66). Before collecting nucleic acid samples, the person in charge can organize a training program to elaborate on the possible changes in the epidemic and the responses to emergencies. This will allow nurses to be better prepared psychologically and relieve their anxiety for the future. To lower the risk of heatstroke among nurses collecting nucleic acid samples, the organizer should provide relevant support materials depending on the weather, such as parasols and big fans when it is hot. Furthermore, nursing managers should implement policies that provide incentives and opportunities for nurses to advance professionally, as well as specific policy support. To improve nurses' sense of self-worth, sense of identity as professionals, and enthusiasm for their work, it is beneficial to improve their publicity work. Nursing management should provide fair shifts, strengthen the backup staff for the nucleic acid collection nurses, and suitably reduce the time required for each task. Nurses are told to reduce the stress and worry experienced by them as a result of their lack of awareness of proper COVID-19 isolation, disinfection, and protection. Nursing managers should actively join the team of nucleic acid sample collection by nurses, establish open communication with nurses, understand their practical needs, and provide necessary assistance and support.

## 7. Conclusion

This study investigated the quality of life, psychological resilience, and social support of nurses who collected nucleic acid samples, explored the relationship among the three, and identified the variables that influence the quality of life of these front-line nurses. According to this study, age, marital status, sleep duration, frequency of exercise, psychological resilience, and social support are all variables that impact nurses' quality of life and should be taken into consideration by both nurses and managers. These findings can provide insights into improving the quality of life of nurses and help nursing managers adopt effective measures accordingly. In this sense, the study contributes to the stability of the nurse team collecting nucleic acid samples and plays a positive role in the prevention and control of the spread of COVID-19.

## Data availability statement

The original contributions presented in the study are included in the article/supplementary material, further inquiries can be directed to the corresponding authors.

## Author contributions

JY, CW, and YuL contributed equally to the research design and drafting manuscript. YuL, HZ, and CH designed and collected the questionnaire. YaL and YangL made statistics and analysis. YZ designed the study and provided guidance on statistical methods. YiL and HL contributed to the final critical revision of the manuscript. All the authors reviewed the final manuscript and agreed to submit it.
